# Introduction of a New Protocol to Limit the Number of Cancelled Elective Orthopaedic Operations Due to Asymptomatic Bacteriuria

**DOI:** 10.7759/cureus.51097

**Published:** 2023-12-26

**Authors:** Noah Taylor, Alexander Jaques, Mohamed Antar, Aashish Raghu, Stephen Tai

**Affiliations:** 1 Surgery, Lister Hospital, Stevenage, GBR; 2 Vascular Surgery, Lister Hospital, Stevenage, GBR; 3 Trauma and Orthopaedics, Lister Hospital, Stevenage, GBR

**Keywords:** quality assessment in healthcare of urinary tract infections, elective orthopaedic surgery, urinalysis testing, surgical site infection (ssi), asymptomatic bacteriuria (asbu)

## Abstract

Background

Asymptomatic bacteriuria (ASB) poses a significant diagnostic dilemma for medical professionals. Current hospital screening protocol determines the likelihood of a positive diagnosis of a urinary tract infection (UTI) based on the results of a bedside urinalysis. ASB, defined as a positive urine culture in the absence of symptoms, can contribute to unnecessary cancellations, poor utilisation of theatre time, and delayed patient care. We present a two-cycle audit proposing a new pathway to addressing ASB in patients awaiting elective orthopaedic surgery, aiming to optimise surgical yield. Our objectives are to identify areas for improvement in our departmental practices with respect to asymptomatic bacteria compared to the published literature. We propose a new protocol targeted to improve our current practices to minimise patient cancellations and optimise theatre utilisation.

Methodology

A total of 78 patients who had an elective orthopaedic procedure cancelled at a large district general hospital offering tertiary orthopaedic services, between two study periods spanning March 2018 to April 2019 and May 2019 to March 2020, were identified from electronic hospital records and theatre management systems. Demographics, procedure details, and reasons for cancellations, including the result of urinalysis and the presence of UTI symptoms were assessed. Our pathway was introduced after the first study period and, subsequently, re-audited to assess adherence to the new protocol and its effect on cancellations.

Results

We identified 78 patients, with a 50:50 male:female split and an average age of 63 (range = 9-90). Of the 33 patients in the first cohort, seven (21.2%) were cancelled due to UTI risk based on positive urinalysis. Of these seven cancellations, one (14.3%) patient reported symptoms of a UTI. The second cohort comprised 45 patients, two (4.4%) of whom were cancelled due to UTI risk based on symptom questionnaire results. These two symptomatic patients along with another two asymptomatic patients (8.8% in total) were found to have positive urinalyses; however, the two asymptomatic patients had their operations cancelled for unrelated reasons.

Conclusions

The study has shown that previously of all patients awaiting elective orthopaedic operations who had their procedures cancelled, 85.7% were cancelled due to ASB. After the introduction of a new protocol focussing on symptoms rather than urinalysis, we estimate that the number of cancelled elective orthopaedic operations has reduced by 71.4%, thereby greatly improving the utilisation of theatre time.

## Introduction

Orthopaedics is the surgical specialty with the second highest activity in the UK, a large proportion of which is made up of elective work [1]. Urinary tract infections (UTIs) are most commonly caused by bacteria and are highly prevalent in the general population. A 2016 study by Tandogdu and Wagenlehner found the global prevalence of UTI to be 0.7% [2]. A bedside urinalysis is routinely used to detect the presence of bacteria in the urine; however, not all patients with bacteriuria are symptomatic. This is termed asymptomatic bacteriuria (ASB). The evidence to suggest that the presence of ASB at the time of surgery is a contributing factor to postsurgical infections is poor [3]. There is also limited evidence to support the treatment of asymptomatic individuals before they undergo this type of surgery [4]. In addition, it is well known that bedside urinalysis is an imperfect indicator of the presence of a UTI, especially in older adults over the age of 65, and their results need to be interpreted carefully with consideration of the clinical presentation [5].

The study was conducted at the Lister Hospital, Stevenage, part of East & North Herts NHS Trust (ENHT), a large district general hospital offering tertiary orthopaedic services. Before April 2019, if patients attending the trust for elective operations were found to have a bedside urinalysis indicative of the presence of an active UTI, their planned procedure was automatically cancelled regardless of whether they were symptomatic. These cancellations represent a substantial waste of clinical time, financial loss, and damage to patient satisfaction. The development of a new protocol to assess patients for UTIs preoperatively would, therefore, be of benefit.

Objectives

This study aimed to evaluate the number of elective orthopaedic operations cancelled due to the risk of UTI during a period with current guidance in use, measuring the proportions of patients who had urinalyses indicative of active UTI and those who were experiencing UTI symptoms. A new pathway which separated patients with symptomatic and asymptomatic bacteriuria into two groups with different managements would then be instigated. Following this, the study would be run again and contrasted with the original data to assess the impact on operative cancellations.

## Materials and methods

This was a single-centre retrospective study assessing two time periods: the first between March 2018 and April 2019 (13.5 months, March 14, 2018, to April 30, 2019), and the second between May 2019 and March 2020 (10.5 months, May 01, 2019, to March 17, 2020). Inclusion criteria were all patients due to be undergoing elective orthopaedic surgery whose operations were cancelled. These procedures ranged from arthroplasties to joint injections. There were no exclusion criteria from this cohort. Data were collected retrospectively from patient notes, CIPTS (theatre management system run by Delian Systems Ltd., no longer in business) and ICE (investigation reporting system) [6]. Data collection parameters included patient demographics (name, age, sex, identification number), procedure planned, date of planned procedure, and reason for and details of cancellation. The same methods were used for both study periods with no discrepancy.

Every patient attending the Lister Hospital for an elective surgery has a bedside urinalysis on the day of admission before their procedure. The original protocol for cancellation of elective operations due to risk of UTI was that any patient with a urinalysis suggestive of active UTI (colloquially known as a “positive urinalysis”) on the day of surgery was cancelled and rebooked, regardless of if they were symptomatic or not. A positive urinalysis had leucocytes and/or nitrites present.

With the proposed new protocol, as well as having a urinalysis on admission, every patient is required to fill out a questionnaire screening for common symptoms of UTI.

The patient questionnaire included the following questions: (1) Do you suffer from burning or stinging when you pass urine? (2) Do you have to pass urine more frequently than usual? (3) Have you noticed blood, discolouration, or cloudiness in your urine? (4) Have you noticed a change of smell in your urine? (5) Do you have any difficulty passing urine? (6) Have you had a confirmed UTI in the last month? (7) Have you had confirmed UTIs in the past?

Patients who report to be experiencing just one or no symptoms of UTI can proceed to undergo surgery as planned, regardless of their urinalysis result. Patients who report to be experiencing two or more of the symptoms on the screening questionnaire have their operations cancelled. Their urine is then sent for culture and the patient is treated with a course of antibiotics as per trust guidelines. Once asymptomatic they can then be reconsidered for surgery.

The new symptoms-based protocol and patient questionnaire were introduced on the morning of May 01, 2019. Before this, cancellations were solely based on urinalysis results. The first and second datasets were analysed retrospectively after the last date of collection from the second cohort (March 17, 2020).

## Results

We analysed data from a total of 78 patients, 39 (50%) males and 39 (50%) females, with an average age of 63 (range = 9-90). There were 33 patients in the first (2018-2019) cohort, 18 (55%) males and 15 (45%) females, with an average age of 61 (range = 16-86). Of these, seven (21.2%) were cancelled due to UTI risk based on a positive urinalysis as per protocol, comprising two (29%) males and five (71%) females, with an average age of 80.4 (range = 71-84). Only one patient, 14.3% of those cancelled due to urinalysis results (3.0% of the cohort), was found to be symptomatic. This patient was an 80-year-old male. The other six (85.7%) patients with a positive urine dip but asymptomatic comprised one (16.7%) male and five (83.3%) females, with an average age of 80.5 (range = 71-84).

There were 45 patients in the second (2019-2020) cohort, 21 (47%) males and 24 (53%) females, with an average age of 65 (range = 9-90). Overall, two patients within this group (4.4% of the cohort), both females aged 50 and 71 (average 60.5), were cancelled due to UTI risk based on reporting symptoms of active infection on the preoperative questionnaire. Another two (4.4%) patients were found to have a positive urinalysis but were asymptomatic; however, these patients’ operations were cancelled due to unrelated reasons. One of these patients was male and the other was female, aged 75 and 81, respectively (average 78.0). Table 1 shows the above data in full.

**Table 1:** Breakdown of proportions of patients cancelled due to UTI risk and of those who had positive urinalyses and/or were symptomatic before and after the introduction of the new protocol. UTI = urinary tract infection; M = male; F = female

Study period	2018–2019	2019–2020
Cohort (all-cause cancellations)	33 [55% M (18), 45% F (15), 61 years (16–86)]	45 [47% M (21), 53% F (24), 65 years (9–90)]
Cancellations due to the risk of UTI	21.2% (7) [29% M (2), 71% F (5), 80.4 years (71–84)]	4.4% (2) [0% M (0), 100% F (2), 60.5 years (50–71)]
Proportion of all-cause cancellations with positive urinalysis	21.2% (7) [29% M (2), 71% F (5), 80.4 years (71–84)]	8.8% (4) [25% M (1), 75% F (3), 69.3 years (50–81)]
Proportion of cancellations due to risk of UTI with negative urinalysis	100% (7) [29% M (2), 71% F (5), 80.4 years (71–84)]	100% (2) [0% M (0), 100% F (2), 60.5 years (50–71)]
Proportion of cancellations due to the risk of UTI reporting symptoms of UTI	14.3% (1) [100% M (1), 0% F (0), 80 years (80–80)]	100% (2) [0% M (0), 100% F (2), 60.5 years (50–71)]

## Discussion

Postoperative infections are a severe and potentially life or limb-threatening risk of any invasive procedure. Orthopaedic surgeries, particularly those involving implanted metalwork, are particularly at risk, and extensive infection prevention measures are taken by orthopaedic teams to minimise their occurrence. Prosthetic joints are at high risk as relatively few individual bacteria either aerosolised pre/perioperatively or seeded postoperatively onto the surface of the prosthesis are required to form a biofilm, a mat of bacteria exceptionally resistant to antibiotic treatment [7]. These infections can be so severe and untreatable that the prosthesis may have to be completely removed, exposing the patient to further operative risk. A 2023 meta-analysis by Mengistu et al. of 49 studies conducted in 39 countries concluded that the global incidence of surgical site infections (SSIs) was between 2.5 and 2.7% [8]. A 2015 meta-analysis by Lamagni et al. regarding the incidence of SSIs following orthopaedic surgery found that the proportion of patients found to have had SSIs at one year post-procedure was 0.7% for knee replacement and 1.0% for hip replacement overall [9]. The same study reported that the rates of revision arthroplasties in the UK due to infection were 23% for knee replacements and 3% and 82% for two-stage hip revisions, respectively.

Many interventions have been applied to current orthopaedic clinical practice to mitigate the risk of postoperative infections. These measures can be categorised into pre, peri, and postoperative measures and include active methicillin-resistant Staphylococcus aureus decontamination, double gloving, sealed theatres, double in-theatre sterilisation, enhanced personal protective equipment, intensive postoperative mobilisation and discharge, rigorous blood glucose control, and extended antibiotic courses [10,11]. An established procedure spanning UK practice is to detect and treat pre-existent infections before proceeding with planned operations. However, the evidence to support distant infections to be a major risk for wound infections is weak. Tande and Patel stated that only seven out of 551 (1.3%) patients with concurrent distant infections during hip or knee arthroplasty were likely to be related to these infections [7]. A commonly accused source of infection is that of the urinary tract, an accusation that Tucci et al. attributed to a small number of studies conducted in the 1970s; however, they alleged that these studies failed to present any convincing evidence that correlate UTIs with prosthetic joint infections [12].

The urinary tract is one of the most common causes of infection [13]. The evidence to point the finger at UTIs as a cause of SSIs, however, is very limited. Cordero-Ampuero et al. alleged that bacteria cultured from surgical wounds are dissimilar to those cultured from the same patients’ preoperative urine samples [4]. The problem of ASB is also a regular point of discourse among healthcare professionals. As mentioned before, it is common practice to postpone orthopaedic surgery based solely on urinalysis results as evidenced in the previous local hospital protocol. Bedside urinalysis tests are a ubiquitous feature of modern hospital investigatory practice. However, their accuracy in detecting infections is known to be nuanced. ASB is more common in women and increases in incidence with age. Luu and Albarillo found the incidence of ASB to be more than 15% in older women, increasing to 50% of those residing in long-term care facilities [14]. With such a high rate of ASB, and limited evidence to support the claim that having a UTI at the time of surgery increases the risk of SSIs, why are patients getting their operations cancelled based on a bedside urinalysis?

As previously mentioned, the cancelling of elective operations has a notably negative effect on service utility and financial and patient welfare outcomes. To run an operating theatre in the UK costs an average of £1,200 per hour [15]. The average UK orthopaedic procedure lasts 81 minutes, and the average number of cases per full-day (9 am to 5 pm) orthopaedic list is 3.8 [16]. This means that every cancelled operation could result in the wastage of 2.1 hours on average of highly skilled healthcare professionals’ time, amounting to an average £2,525 loss of income. Putting this into the context of the current NHS financial and staffing issues, it appears as an unacceptable and preventable failure.

As there is no data available for patients who had a urinalysis suggestive of UTI who were not cancelled, we cannot get an absolute measure of the number of operations that the new protocol prevented from being cancelled due to UTI risk. However, we can estimate this by extrapolating from the first cohort data, assuming that the proportion of patients with positive urinalyses who are actually symptomatic (14.3%) is the same and subtracting the proportion of patients with positive urinalyses who would have had their operations cancelled for unrelated reasons (equal to that of the proportion of cancellations in the second cohort who are symptomatic). We can, therefore, conclude that the new protocol has reduced the number of cancelled elective orthopaedic operations due to UTI risk by an estimated 71.4%. We can also conclude that we have reduced the number of cancelled orthopaedic operations due to ASB by 100%.

Recognising the limits of this study, we recommend that another cycle be performed to include patients whose operations were not cancelled but had positive urinalyses. This will allow us to get a definite value of the total number of ASB cases to quantify the proportion of cases prevented from being cancelled unnecessarily after the introduction of the new protocol. This was also a single-centre study. Additional centres of similar characteristics may be added in the future to improve reliability.

## Conclusions

On comparing the two cohorts, 85.7% of the patients in the first cohort with positive urine dips (18.2% of the total cohort) would not have been cancelled if the new protocol had been used. In the second cycle, we noted that 100% of the patients with positive urinalyses were symptomatic. Although the number of all-cause cancellations was higher in the second cohort, this is indicative that no patients had their operations cancelled due to ASB. We can, therefore, conclude that the introduction of this new symptoms-based protocol has reduced the number of elective orthopaedic operations cancelled unnecessarily due to ASB by an estimated 71.4%.
